# Effectiveness and content analysis of interventions to enhance medication adherence in hypertension: a systematic review and meta-analysis protocol

**DOI:** 10.1186/s13643-016-0278-5

**Published:** 2016-06-07

**Authors:** Eimear C. Morrissey, Hannah Durand, Robby Nieuwlaat, Tamara Navarro, R. Brian Haynes, Jane C. Walsh, Gerard J. Molloy

**Affiliations:** School of Psychology, National University of Ireland, Galway, Ireland; Department of Clinical Epidemiology and Biostatistics, McMaster University, Hamilton, Ontario Canada

**Keywords:** Hypertension, Medication adherence, Blood pressure, Compliance

## Abstract

**Background:**

Hypertension control through pharmacological treatment has led to substantial benefits in the prevention of morbidity and mortality from cardiovascular diseases. However, evidence from a number of studies suggests that as many as 50 to 80 % of patients treated for hypertension have low adherence to their treatment regimen. The objective of this systematic review is to evaluate the effectiveness of medication adherence interventions for hypertension. In addition, we aim to explore what barriers and facilitators in the interventions may have been targeted and how these might be related to the effect size on blood pressure (BP).

**Methods:**

This review is a hypertension-specific update to the previous Cochrane Review by Nieuwlaat et al. (2014) on interventions to enhance medication adherence. A systematic literature search will be carried out, and two authors will independently screen titles and abstracts for their eligibility for inclusion and independently extract data from the selected studies and assess the methodological quality using the Cochrane Collaboration Risk of Bias Tool. A meta-analysis will be conducted, and additionally, theoretical factors in interventions will be identified using the Theoretical Domains Framework.

**Discussion:**

This review will generate new information by quantitatively evaluating the effectiveness of adherence interventions for hypertension and potentially identify which theoretical domains are associated with more effective interventions and which domains have not been the subject of intervention development.

**Systematic review registration:**

PROSPERO CRD42016033358

**Electronic supplementary material:**

The online version of this article (doi:10.1186/s13643-016-0278-5) contains supplementary material, which is available to authorized users.

## Background

### Rationale

#### Description of the condition

Hypertension, also known as high or raised blood pressure (BP), is a condition in which the blood vessels have persistently raised pressure. Epidemiologic studies demonstrate that cardiovascular disease events (e.g. stroke, heart failure and coronary heart disease) are associated with hypertension [1, 2]. A systematic review by Kearney et al. [3] found it to be an important risk factor for more serious conditions that carry greater risk of disability and death (primarily cardiovascular and cerebrovascular events) and the single most important modifiable risk factor for stroke and myocardial infarction in both developed and developing countries. It is estimated that hypertension affects one billion people worldwide [4].

Hypertension control through pharmacological treatment has led to substantial benefits in the prevention of morbidity and mortality from cardiovascular disease [5]. A Cochrane review conducted by Musini et al. [6] was an assessment of all the trials of blood pressure lowering therapy in people with hypertension aged 60 years and over and found that these treatments reduced death, strokes and heart attack. However, despite the efficacy of antihypertensive agents, there is a significant problem of non-adherence to these medications in those diagnosed with hypertension; therefore, the effectiveness of current treatment is limited.

The World Health Organization (WHO) defines adherence to long-term therapy as ‘the extent to which a persons behaviour – taking medication, following a diet and/or executing lifestyle changes – corresponds with agreed recommendations from a health care provider’ [7]. High adherence (defined as medication possession ratio of 80 to 100 %) to hypertensive medications is associated with higher odds of blood pressure control compared with those with medium or low levels of adherence [8]. Evidence from a number of studies suggests that as many as 50 to 80 % of patients prescribed pharmacological antihypertensive therapy have low adherence to their treatment regimen [9]. Vrijens et al. [10] used medication event monitor system (MEMS) data to measure adherence to antihypertensive medications and found that about half of all patients prescribed the medications stopped taking them within one year of the initial prescription. They also found that on any one day, 10 % of patients omitted their scheduled dose of medication. According to the WHO, this lack of adherence to antihypertensive medication is the most important cause of failure to achieve BP control [7].

#### Description of the interventions

Interventions for enhancing medication adherence in hypertension can have multiple components, such as education around the condition and the importance of adherence, skills development, combination pills, provision of practical support and self-monitoring of blood pressure. These interventions have had mixed results (e.g. [11, 12]). Conn et al. [13] conducted a meta-analysis combining randomised and non-randomised studies that targeted anti-HT medication adherence improvement and found that 112 intervention vs control group comparisons had a standardised mean difference effect size of 0.3 (SD 0.079) on adherence. However, there was significant heterogeneity across studies (*I*^2^ = 87 %), which can be explained by heterogeneity in samples, designs and measures used to assess adherence.

#### How the intervention might work

Because interventions for adherence in hypertension can have very varied and often multiple components, it can be difficult to ascertain which factors are causing the intervention to work or are masking effective components. Behavioural theory can be used to gain an understanding of the effects of the behaviour change intervention. However, papers rarely report the explicit use of theory despite interventions almost certainly involving at least an implicit idea of what factors to address to instigate change [14]. This means that even if an intervention is successful, it is difficult to understand the behaviour change processes responsible and therefore to inform future intervention design, refinement and application. Consequently, it may be of benefit to retrospectively identify which barriers and facilitators adherence interventions report targeting and the extent to which such factors map onto pre-existing theoretical factors. In order to capture the potential range of possible targeted factors, a sufficiently broad framework of theoretical factors is required. The Theoretical Domains Framework (TDF) is an integrative framework which was developed and validated to summarise the range of psychological theory underpinning behaviour change into distinct factors [15, 16]. By applying the TDF to adherence interventions, it may be possible to explore which theoretical domains modify effect size (e.g. [14, 17]).

#### Why it is important to do this review

There are two reasons why it is important to do this review. Firstly, it is a hypertension-specific update to the previous Cochrane review by Nieuwlaat et al. [18] on interventions to enhance medication adherence. This was an extremely large review, encompassing all medical conditions. Due to the heterogeneity of outcomes across conditions, it was not feasible to conduct a meta-analysis as part of the research. As this study is focusing specifically on hypertension, it is anticipated that most of the included interventions will have a common outcome of change in blood pressure in addition to the outcome of medication adherence. This will allow quantitative synthesis and additional analysis that was not possible in the previous review. Secondly, to the best of our knowledge, identifying whether the targeting of particular theoretical domains in the interventions is associated with greater effect sizes in adherence interventions in hypertension has not been done previously. Recent studies have attempted to synthesise existing evidence using a similar general approach (e.g. Little et al. [14] used the TDF within a systematic review of interventions to improve quality of care in post-fracture investigation.) This will be a valuable addition to the literature as it may inform future intervention design.

### Objectives

The objective of this review is to evaluate the effectiveness of adherence interventions for hypertension and explore which specific barriers and facilitators the interventions may have been targeting and how this tailored approach might be related to the effect size on blood pressure (primary outcome) and medication adherence.

## Methods

### Eligibility criteria

This protocol has been developed in line with the Preferred Reporting Items for Systematic Review and Meta-Analysis Protocols (PRISMA-P) statement [19] (see Additional file 1). The systematic review and meta-analysis will be conducted and reported in accordance with the PRISMA statement [20] and is registered with the PROSPERO (International Prospective Register of Systematic Reviews) database (registration number CRD42016033358).

#### Types of studies

The systematic review is a condition-specific update to the large review by [18] on interventions to enhance medication adherence which searched for studies until January 2013. This review will include randomised controlled trials (RCTs) that provide unconfounded tests of interventions expected to enhance adherence. Studies will be included regardless of treatment intensity or duration, mode of treatment delivery or medium of treatment.

#### Types of participants

The participants will be patients who were prescribed medication for hypertension.

#### Types of interventions

Interventions of any sort intended to affect adherence with prescribed, self-administered medication for the treatment of hypertension.

#### Types of outcome measures

The primary outcome will be the change in blood pressure (SBP, DBP, or both) at 6 months compared with baseline readings taken prior to the intervention. The change in BP may be reported as a dichotomous (% with positive/negative BP change) or continuous variable (mean change in BP values). The primary outcome will be compared between treatment groups, i.e. the difference between groups regarding the change in BP.

The secondary outcome will be medication adherence measured by at least one of the following: self-report, pill count, pharmacy refill records and electronic medication monitors (MEMS).

Interventions will have to include both outcomes to be included.

#### Follow-up completion

Regarding follow-up completion, studies will need to have at least 80 % follow-up during at least 6 months. The 80 % data completion is required for both blood pressure and adherence outcomes.

### Information sources

#### Search methods for identification of studies

We will search *The Cochrane Library* including CENTRAL, MEDLINE, EMBASE, PsycINFO, CINAHL and Sociological Abstracts. This database search will be a hypertension-specific update on previous searches that were undertaken on 1 September 1993, 12 December 1993, 1 June 1994, 30 June 1995, 31 July 1998, 15 August 2001, 30 September 2004, 1 February 2007 and 11 January 2013. We will search new publications since 11 December 2012, that is, having a 1-month overlap with the previous search. All databases were originally searched from their start date. Ongoing trials will be identified by checking trials and protocols published in relevant databases of current ongoing clinical research studies, specifically World Health Organization International Clinical Trials Registry Platform and ClinicalTrials.gov.

### Search strategy

The search filters for each database can be seen in Appendix 1. We will check articles cited in reviews and original studies of patient adherence in hypertension. We will contact authors of included RCTs to identify additional studies.

## Study records

### Data management

We will use a web-based data management system, developed by the Health Information Research Unit at McMaster University to facilitate screening, data extraction, adjudication of disagreements, author review and confirmation of data, production of data tables and production of data files for future research use. This system has been successfully used in conducting and completing several large, complex systematic reviews.

### Selection process

We will re-assess all RCTs on hypertension included in the 2014 update for eligibility to carry over into the current update. Retrieved citations from the updated search will enter a first screening stage. Based on the title and abstract, studies will move to the second screening stage if they meet all five eligibility criteria or if there is uncertainty about their eligibility. In the second screening stage, assessment of the full text will determine if studies will be included on the review. At both screening stages, two independent review authors (EM and HD) will assess eligibility and an adjudicator (TN) will resolve disagreements. We will record reasons for excluding citations in the second screening stage and report these in a PRISMA flow chart.

## Data collection and analysis

### Data collection process

We will import data from the 2014 review into the update database and check the data for accuracy. Extracted data includes items as provided in the tables from the previous review [18]: the ‘characteristics of included studies’ table (i.e. methods, participants, interventions, outcome, additional notes pertaining to any one of the aforementioned items, detailed assessment of risk of bias), the ‘adherence and outcome’ table (i.e. intervention, control, effect on adherence outcome, effect on clinical outcome) and risk of bias summary. We will extract the same items for the new included studies. Two review authors (EM and HD) will independently extract all new data and an adjudicator (TN) will resolve disagreements. We will contact primary or corresponding authors of all included RCTs to confirm extracted data and provide missing data.

The TDF domains that appeared to be targeted by the interventions and within the control groups will be identified and coded independently by two reviewers (EM and GM), using a data extraction form designed for the purpose. This will be based on similar work by Little et al. [14]. We will use domains as well as constructs within domains to inform coding decisions within domains, using construct definitions as described by Cane et al. [16]. The data extraction form will be tested on one included study. The coding of each domain will be supported by evidence from the text. Inter-rater reliability will be calculated prior to resolving discrepancies. Discrepancies will be discussed until 100 % agreement is achieved.

### Assessment of risk of bias in included studies

Two authors (EM and HD) will independently use the Cochrane ‘Risk of bias’ tool described in *The Cochrane Handbook for Systematic Reviews of Interventions* [21] to assess randomisation procedures, bias, allocation, outcome assessors, reporting of findings and losses to follow-up.

### Measures of treatment effect

For the primary outcome of BP, we will report mean differences between groups and the 95 % confidence intervals (95 % CI).

For the secondary outcome of medication adherence, it is likely that different measurement tools will have been used and so we will calculate the standardised mean difference and the 95 % CI for continuous data.

Where no standard deviations are reported, we will calculate the standard deviations using the methods described in *The Cochrane Handbook for Systematic Reviews of Interventions* [21].

### Unit of analysis issues

In studies where more than one intervention group is contained in one comparator arm, we will include both interventions (providing they are relevant to the review) and will split the number of participants between the two groups accordingly. Where cluster RCTs are included for analysis we will use appropriate statistical analysis methods to account for the cluster effect as described in *The Cochrane Handbook for Systematic Reviews of Interventions* [21].

### Assessment of heterogeneity

We will use the *I*^2^ statistic and the Chi^2^ test to assess heterogeneity as described *in The Cochrane Handbook for Systematic Reviews of Interventions* [21]. We will initially carry out a qualitative analysis of the heterogeneity of the study groups by study design e.g. number of randomised groups and data collection methodology e.g. measurement strategy to assess adherence. We will quantitatively analyse suitable data subsequently using regression analysis.

### Assessment of reporting bias

We will assess reporting bias initially by a visual inspection of funnel plots and use of appropriate statistical tests as described in *The Cochrane Handbook for Systematic Reviews of Interventions* [21]. Some studies may not report absolute blood pressure changes, but rather proportional changes from the baseline measurement or another parameter. In these cases, we will also consider reporting bias.

### Data synthesis

Data will be pooled and analysed where appropriate and feasible. We will analyse each outcome measure separately, calculate intervention effects and express them as relative risks with 95 % confidence intervals for dichotomous data and as mean differences and standardised mean differences (if required) with 95 % confidence intervals for continuous data. We will give consideration to the use of random-effects models that can be incorporated into the statistical analysis if substantive statistical heterogeneity is identified. In addition, we will use subgroup analyses to assess heterogeneity (see below).

The relationship between the number of different domains coded and the effect size of the intervention will be explored using Pearson correlations (two-tailed). This analysis will be based on similar work by Little et al. [14]. The maximum possible number of different domains coded will be 14 (the number of TDF domains). The number of different domains coded in the control group will be subtracted from the number of different domains coded in the intervention group. A sensitivity analysis will be performed in which the subtraction of control groups was not done, to examine the effect of the subtraction on the result. The analysis will also explore the potential for weighting domains according to the frequency of which the domain was targeted. If no significant difference emerges, the primary approach to data synthesis will be descriptive e.g. identifying the proportion of studies that target specific domains.

### Subgroup analysis

We anticipate that different categories of interventions will be used by the included studies, including context of the intervention delivery, technological interventions, combination pill and service provision interventions. If appropriate, we will use subgroup analysis to categorise these interventions and to explore heterogeneity. Similarly, some studies may only target low adherers while others target all hypertension patients. If numbers allow, subgroup analysis will also be conducted here. If feasible, similar patient sub-group analysis will also be conducted on age and gender.

## Discussion

This proposed review will add to the literature in several ways. Due to population growth, ageing and behavioural risk factors, such as unhealthy diet, harmful use of alcohol, lack of physical activity, excess weight and exposure to persistent stress, there has been a significant growth in the incidence of hypertension; the number of people with hypertension rose from 600 million in 1980 to 1 billion in 2008 [22]. Issues around the control and management of hypertension are becoming increasingly pertinent, in high-income countries and even more so in low- and middle-income countries.

As previously discussed, interventions for medication adherence in hypertension tend to be multi-factorial and there is no ‘one’ recommended strategy for enhancing adherence [18]. It is possible that by mapping intervention components to the TDF we may be able to determine which theoretical factors are associated with larger effect sizes. A systematic review, also using the TDF, by Khatibet al. [23] examined patient and healthcare provider barriers to hypertension awareness, treatment and follow-up. While this analysis of quantitative and qualitative studies provides valuable information, it is possible that self-reported barriers are not always the critical determinants of non-adherence, and therefore, interventions targeting these factors may not be effective. This current review will be an important follow-up on to this, as we will be able to see if the barriers and facilitators reported by the patients and providers are the same barriers and facilitators that have been targeted in intervention RCTs and which intervention targets are associated with larger effect sizes. This will have a stronger potential to inform future intervention development and clinical practice in the area.

There are limitations to retrospectively coding interventions. It is likely that some of the interventions will not employ or fail to report explicit use of theory, as seen in a similar systematic review on adherence by Holmeset al. [24]. This means that coding will have to be based on inference from the text. This may also be challenging as preliminary review of some of the relevant interventions show that some of them are lacking comprehensive descriptions of the intervention content. However, all authors will be contacted and asked to provide more information and intervention manuals where possible. Also, coding will be carried out by two reviewers to allow for better sensitivity and enhanced reliability of the theoretical content. Another limitation of this review is that it only includes RCTs. This is the highest quality evidence, but large population and policy interventions are typically not tested in RCTs and thus not captured. As organizational/policy interventions are part of a wider approach to behaviour change intervention, our focus on RCTs where individuals or clusters are randomised to treatments is appropriate. Using a more heterogenous set of study designs and intervention approaches would lead to a less coherent and defensible synthesis of study findings. This review will provide the highest quality evidence for specific intervention techniques that should be part of larger programmes of policies, if proven effective. A final limitation is the observational nature of this work, any association found between domains and effect size will be correlational and as such, causation cannot be inferred.

This review will be of interest to researchers, health professionals, healthcare commissioners and patient groups. It will generate new information by quantitatively evaluating the effectiveness of adherence interventions for hypertension and potentially identify which theoretical domains are associated with more effective interventions and which domains have not been the subject of intervention development.

## Abbreviations

BP, blood pressure; DBP, diastolic blood pressure; EM, Eimear Morrissey; GM, Gerard Molloy; HD, Hannah Durand; MEMS, medication event monitoring system; PRISMA, preferred reporting items for systematic reviews and meta-analyses; PROSPERO, international prospective register of systematic reviews; RCT, randomised controlled trial; SBP, systolic blood pressure; TDF, theoretical domains framework; TN, Tamara Navarro; WHO, World Health Organization

## Appendix 1

MEDLINE search filters used in this update using the Ovid interface:

1. ((exp patient compliance/ OR (patient adj compliance).tw. OR (patient adj adherence).tw. OR (medication adj compliance).tw. OR (medication adj adherence).tw.) AND ((clinical trial OR random:).mp. OR tu.xs.)) NOT ((qualitative OR retrospective OR mice OR rat OR rats).tw. OR editorial.pt. OR letter.pt. OR comment.pt.) NOT (animals NOT humans).sh. (Note: clinical trial.mp. picks up clinical trial.pt.)
2. ((random: OR control:).mp. AND (exp patient compliance/ OR patient dropouts/ OR psychotherapy/ OR treatment refusal/ OR patient education/ OR regimen:.tw.) AND (intervention: OR outcome:).tw. AND (medicat:.tw. OR drug therapy/)) NOT ((qualitative OR retrospective OR mice OR rat OR rats).tw. OR editorial.pt. OR letter.pt. OR comment.pt.) NOT (animals NOT humans).sh.
3. ((exp hypertension/ OR (blood adj pressure).ti. OR hypertens$.ti OR/1-3))

CINAHL search filters used in this update using the EBSCO interface:

1. MH patient compliance + OR TI ‘patient compliance’ OR AB ‘patient compliance’ OR TI ‘patient adherence’ OR AB ‘patient adherence’ OR TI ‘medication compliance’ OR AB ‘medication compliance’ OR TI ‘medication adherence’ OR AB ‘medication adherence’ NOT PT editorial or PT letter or TI qualitative or AB qualitative or TI retrospective or AB retrospective or TI mice or AB mice or TI rat or AB rat or TI rats or AB rats (limited by Clinical Queries therapy sensitive search filter and date—2007 to 2012 )
2. MH patient compliance OR MH medication compliance OR MH patient dropouts OR MH treatment refusal OR MH patient education OR TI psychotherapy OR AB psychotherapy AND TX ( (random* OR control*) ) AND TX ( (medicat* OR drug therapy) ) NOT PT editorial or PT letter or TI qualitative or AB qualitative or TI retrospective or AB retrospective or TI mice or AB mice or TI rat or AB rat or TI rats or AB rats
3. MH hypertension OR TI hypertension OR AB hypertension OR MH ‘blood pressure’ OR TI ‘blood pressure’ OR AB ‘blood pressure’ NOT PT editorial or PT letter or TI qualitative or AB qualitative or TI retrospective or AB retrospective or TI mice or AB mice or TI rat or AB rat or TI rats or AB rats

EMBASE search filter used in this update using the Ovid interface: (random: or control:).mp. AND (patient compliance or patient dropouts or illness behavior or psychotherapy or treatment refusal or patient education or regimen:).mp. AND(intervention: or outcome: or treatment outcome).mp. AND(medicat: or drug therapy).mp. AND (clinical trial or controlled study or randomised controlled trial).mp. AND (hypertension or hypertens$ or blood pressure).mp.

PsycINFO search filter used in this update using the Ovid interface: (((control: or random:).tw. or exp treatment/) and (adherence or compliance or noncompliance or dropouts or patient education).mp. and (drug therapy or drug or medicat: or treatment or regimen).mp. and (intervention or outcomes or treatment outcomes).mp.) not (qualitative or retrospective or mice or rat or rats).tw. AND (hypertension or hypertens$ or blood pressure).mp.

The Cochrane Library search filter used in this update (http://onlinelibrary.wiley.com/cochranelibrary/search/):

((random*) AND (complian* or adheren* or pharmacotherapy or regimen* or educat*) AND (medicat*) AND (hypertension or hypertens$ or blood pressure) (Note: Searched using all text tag; Search by product: Cochrane Database of Systematic Reviews, DARE (Other Reviews), Central; Search by record: all)

Sociological Abstracts search filter used in this update using the ProQuest interface (http://search.proquest.com/socabs/advanced): ((patient or treatment or dropouts) AND (clinical trials or control) AND (drugs or medicine or medication) AND (hypertension or hypertens$ or blood pressure) (Searched using all fields; all(medication) retrieves su(medication adherence)

ClinicalTrials.gov search filter used in this update (http://clinicaltrials.gov/ct2/search/advanced):

‘patient compliance’ OR ‘patient adherence’ OR ‘medication compliance’ OR ‘medication adherence’ AND ‘hypertension’ OR ‘blood pressure’ | Closed Studies | Studies With Results |

International Clinical Trials Registry Platform search filter used in this update (http://apps.who.int/trialsearch/AdvSearch.aspx): patient compliance or patient adherence or medication compliance or medication adherence and hypertension or blood pressure (Note: recruitment status all used)

## Additional file

Additional file 1: A populated PRISMA-P checklist. (DOC 80 kb)
