# Erratum to: Effectiveness and content analysis of interventions to enhance medication adherence in hypertension: a systematic review and meta-analysis protocol

**DOI:** 10.1186/s13643-017-0429-3

**Published:** 2017-02-17

**Authors:** Eimear C. Morrissey, Hannah Durand, Robby Nieuwlaat, Tamara Navarro, R. Brian Haynes, Jane C. Walsh, Gerard J. Molloy

**Affiliations:** 1 School of Psychology, National University of Ireland, Galway, Ireland; 2 Department of Clinical Epidemiology and Biostatistics, McMaster University, Hamilton, Ontario Canada

## Erratum

Upon publication of the article [1], it was noticed that there was an error in Appendix 1. The 3^rd^ line should read as:

(exp hypertension/ OR (blood adj pressure).ti. OR hypertens$.ti.) AND (OR/1-2)
